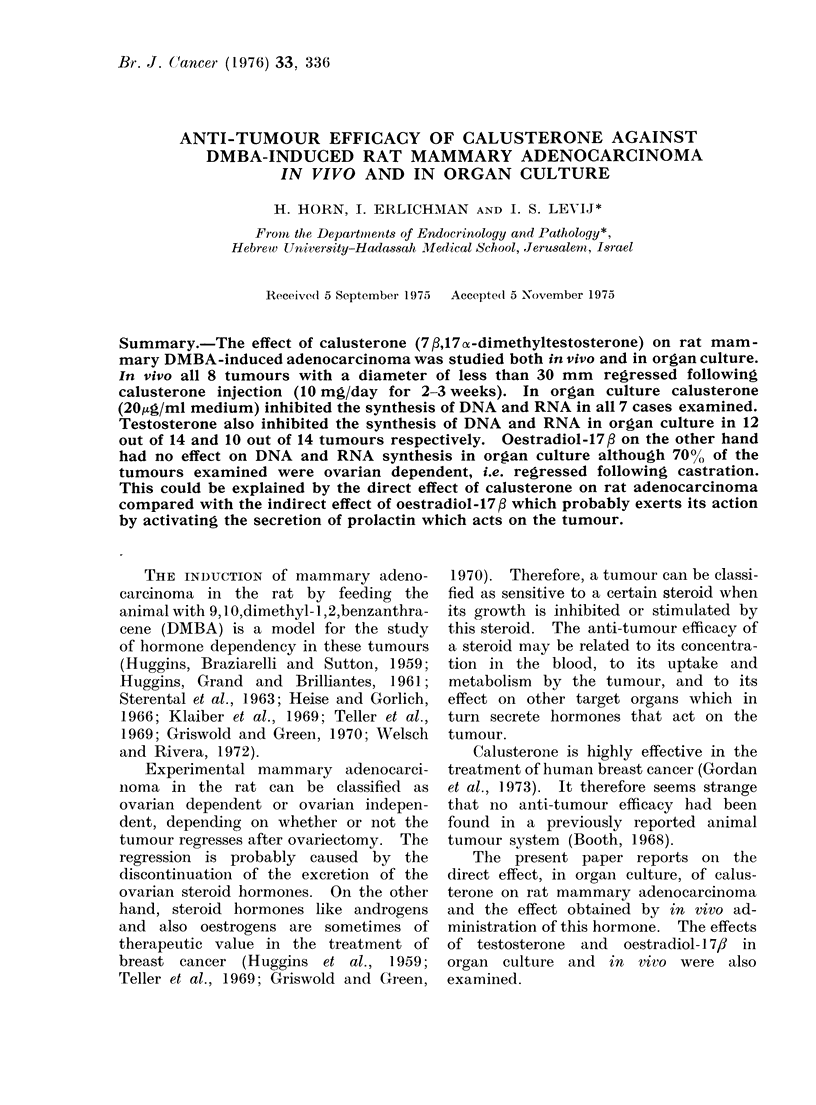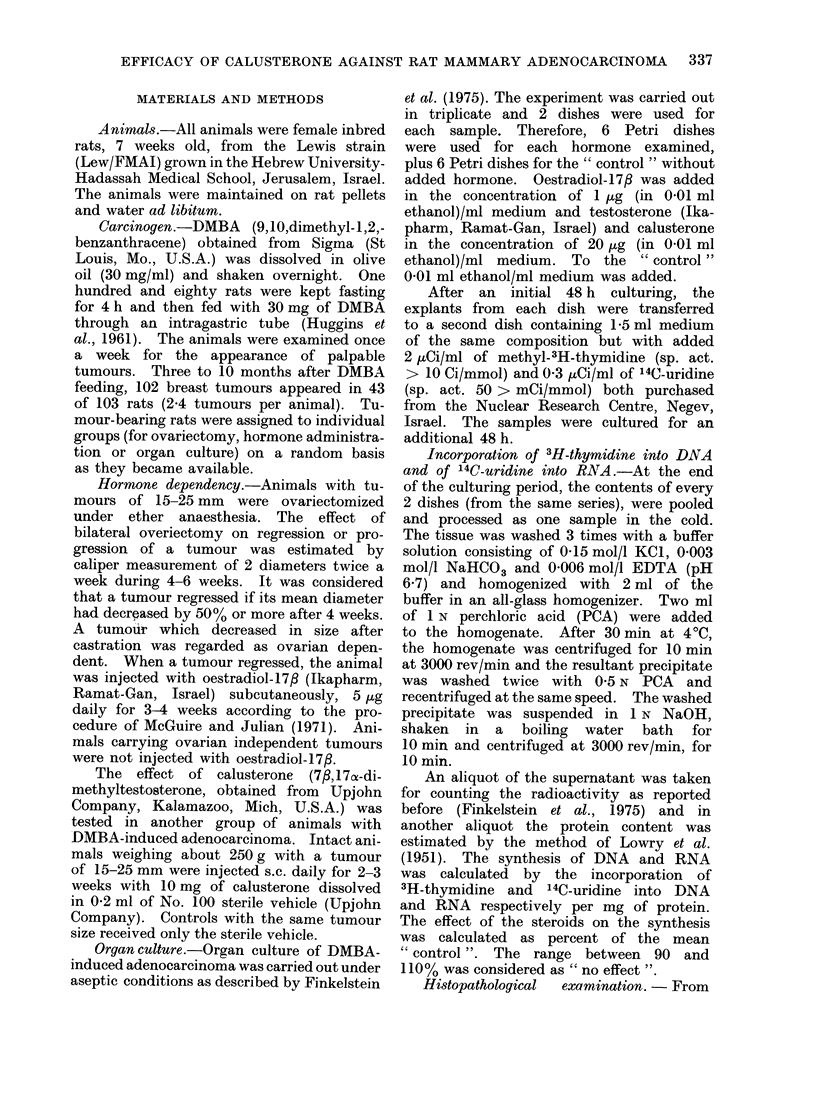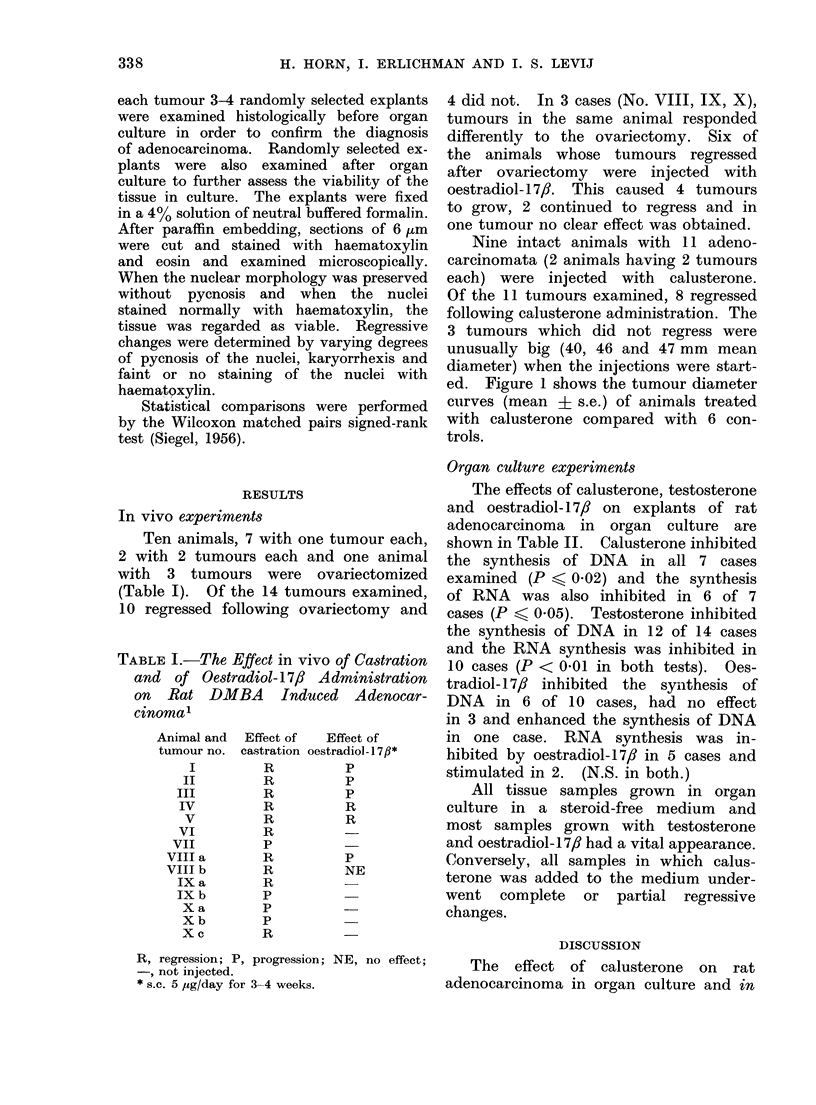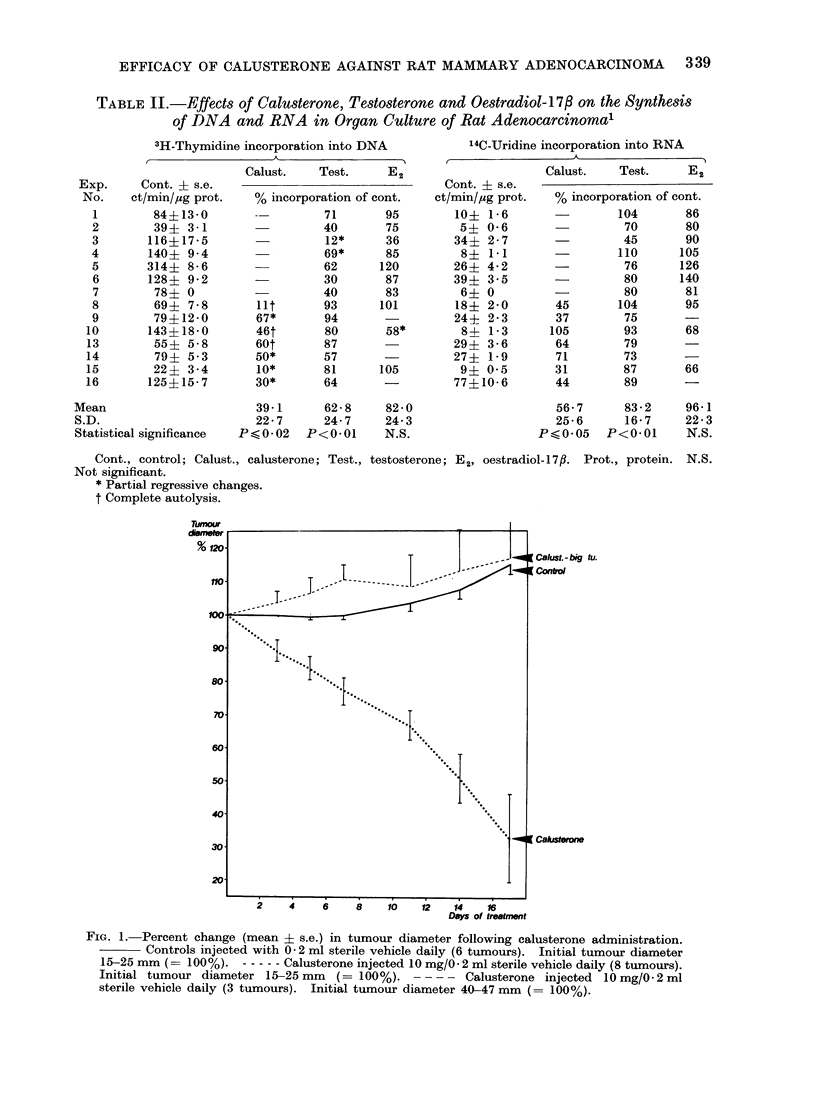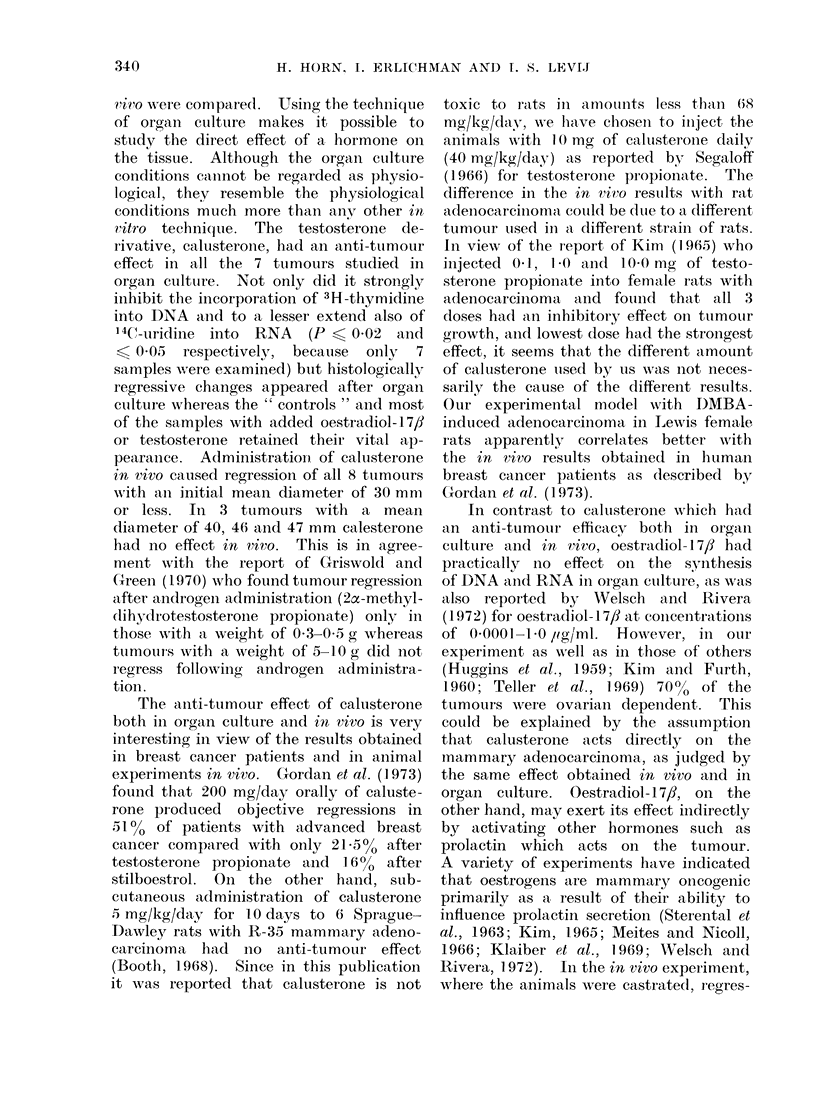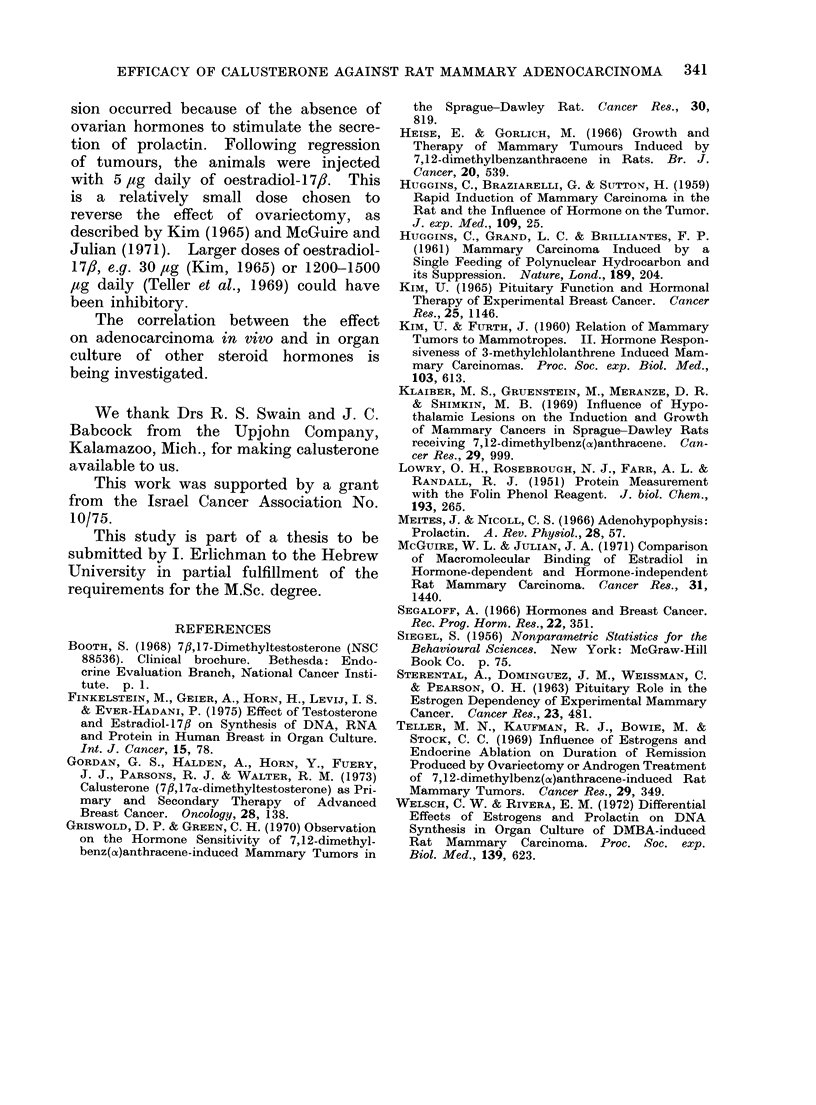# Anti-tumour efficacy of calusterone against DMBA-induced rat mammary adenocarcinoma in vivo and in organ culture.

**DOI:** 10.1038/bjc.1976.48

**Published:** 1976-03

**Authors:** H. Horn, I. Erlichman, I. S. Levij

## Abstract

The effect of calusterone (7beta,17alpha-dimethyltestosterone) on rat mammary DMBA-induced adenocarcinoma was studied both in vivo and in organ culture. In vivo all 8 tumours with a diameter of less than 30 mm regressed following calusterone injection (10 mg/day for 2-3 weeks). In organ culture calusterone (20 mug/ml medium) inhibited the synthesis of DNA and RNA in all 7 cases examined. Testosterone also inhibited the synthesis of DNA and RNA in organ culture in 12 out of 14 and 10 out of 14 tumours respectively. Oestradiol-17beta on the other hand had no effect on DNA and RNA synthesis in organ culture although 70% of the tumours examined were ovarian dependent, i.e. regressed following castration. This could be explained by the direct effect of calusterone on rat adenocarcinoma compared with the indirect effect of oestradiol-17beta which probably exerts its action by activating the secretion of prolactin which acts on the tumour.


Br. J. Cancer (1976) 33, 336

### ANTI-TUMOUR EFFICACY OF CALUSTERONE AGAINST DMBA-INDUCED RAT MAMMARY ADENOCARCINOMA *IN VIVO* AND IN ORGAN CULTURE

H. HORN, I. ERLICHMAN AND I. S. LEVIJ*

*From the Departments of Endocrinology and Pathology*, Hebrew University–Hadassah Medical School, Jerusalem, Israel*

Received 5 September 1975 Accepted 5 November 1975

**Summary.—The effect of calusterone (7β,17α-dimethyltestosterone) on rat mammary DMBA-induced adenocarcinoma was studied both *in vivo* and in organ culture. *In vivo* all 8 tumours with a diameter of less than 30 mm regressed following calusterone injection (10 mg/day for 2–3 weeks). In organ culture calusterone (20μg/ml medium) inhibited the synthesis of DNA and RNA in all 7 cases examined. Testosterone also inhibited the synthesis of DNA and RNA in organ culture in 12 out of 14 and 10 out of 14 tumours respectively. Oestradiol-17β on the other hand had no effect on DNA and RNA synthesis in organ culture although 70% of the tumours examined were ovarian dependent, *i.e.* regressed following castration. This could be explained by the direct effect of calusterone on rat adenocarcinoma compared with the indirect effect of oestradiol-17β which probably exerts its action by activating the secretion of prolactin which acts on the tumour.**

THE INDUCTION of mammary adenocarcinoma in the rat by feeding the animal with 9,10,dimethyl-1,2,benzanthracene (DMBA) is a model for the study of hormone dependency in these tumours (Huggins, Braziarelli and Sutton, 1959; Huggins, Grand and Brilliantes, 1961; Sterental *et al.*, 1963; Heise and Gorlich, 1966; Klaiber *et al.*, 1969; Teller *et al.*, 1969; Griswold and Green, 1970; Welsch and Rivera, 1972).

Experimental mammary adenocarcinoma in the rat can be classified as ovarian dependent or ovarian independent, depending on whether or not the tumour regresses after ovariectomy. The regression is probably caused by the discontinuation of the excretion of the ovarian steroid hormones. On the other hand, steroid hormones like androgens and also oestrogens are sometimes of therapeutic value in the treatment of breast cancer (Huggins *et al.*, 1959; Teller *et al.*, 1969; Griswold and Green, 1970). Therefore, a tumour can be classified as sensitive to a certain steroid when its growth is inhibited or stimulated by this steroid. The anti-tumour efficacy of a steroid may be related to its concentration in the blood, to its uptake and metabolism by the tumour, and to its effect on other target organs which in turn secrete hormones that act on the tumour.

Calusterone is highly effective in the treatment of human breast cancer (Gordan *et al.*, 1973). It therefore seems strange that no anti-tumour efficacy had been found in a previously reported animal tumour system (Booth, 1968).

The present paper reports on the direct effect, in organ culture, of calusterone on rat mammary adenocarcinoma and the effect obtained by *in vivo* administration of this hormone. The effects of testosterone and oestradiol-17β in organ culture and *in vivo* were also examined.

### MATERIALS AND METHODS

*Animals.*—All animals were female inbred rats, 7 weeks old, from the Lewis strain (Lew/FMAI) grown in the Hebrew University-Hadassah Medical School, Jerusalem, Israel. The animals were maintained on rat pellets and water *ad libitum*.

*Carcinogen.*—DMBA (9,10,dimethyl-1,2,-benzanthracene) obtained from Sigma (St Louis, Mo., U.S.A.) was dissolved in olive oil (30 mg/ml) and shaken overnight. One hundred and eighty rats were kept fasting for 4 h and then fed with 30 mg of DMBA through an intragastric tube (Huggins *et al.*, 1961). The animals were examined once a week for the appearance of palpable tumours. Three to 10 months after DMBA feeding, 102 breast tumours appeared in 43 of 103 rats (2·4 tumours per animal). Tumour-bearing rats were assigned to individual groups (for ovariectomy, hormone administration or organ culture) on a random basis as they became available.

*Hormone dependency.*—Animals with tumours of 15–25 mm were ovariectomized under ether anaesthesia. The effect of bilateral overiectomy on regression or progression of a tumour was estimated by caliper measurement of 2 diameters twice a week during 4–6 weeks. It was considered that a tumour regressed if its mean diameter had decreased by 50% or more after 4 weeks. A tumour which decreased in size after castration was regarded as ovarian dependent. When a tumour regressed, the animal was injected with oestradiol-17$\beta$ (Ikapharm, Ramat-Gan, Israel) subcutaneously, 5 $\mu$g daily for 3–4 weeks according to the procedure of McGuire and Julian (1971). Animals carrying ovarian independent tumours were not injected with oestradiol-17$\beta$.

The effect of calusterone (7$\beta$,17$\alpha$-dimethyltestosterone, obtained from Upjohn Company, Kalamazoo, Mich, U.S.A.) was tested in another group of animals with DMBA-induced adenocarcinoma. Intact animals weighing about 250 g with a tumour of 15–25 mm were injected s.c. daily for 2–3 weeks with 10 mg of calusterone dissolved in 0·2 ml of No. 100 sterile vehicle (Upjohn Company). Controls with the same tumour size received only the sterile vehicle.

*Organ culture.*—Organ culture of DMBA-induced adenocarcinoma was carried out under aseptic conditions as described by Finkelstein *et al.* (1975). The experiment was carried out in triplicate and 2 dishes were used for each sample. Therefore, 6 Petri dishes were used for each hormone examined, plus 6 Petri dishes for the " control " without added hormone. Oestradiol-17$\beta$ was added in the concentration of 1 $\mu$g (in 0·01 ml ethanol)/ml medium and testosterone (Ikapharm, Ramat-Gan, Israel) and calusterone in the concentration of 20 $\mu$g (in 0·01 ml ethanol)/ml medium. To the " control " 0·01 ml ethanol/ml medium was added.

After an initial 48 h culturing, the explants from each dish were transferred to a second dish containing 1·5 ml medium of the same composition but with added 2 $\mu$Ci/ml of methyl-$^{3}$H-thymidine (sp. act. $>$ 10 Ci/mmol) and 0·3 $\mu$Ci/ml of $^{14}$C-uridine (sp. act. 50 $>$ mCi/mmol) both purchased from the Nuclear Research Centre, Negev, Israel. The samples were cultured for an additional 48 h.

*Incorporation of $^{3}$H-thymidine into DNA and of $^{14}$C-uridine into RNA.*—At the end of the culturing period, the contents of every 2 dishes (from the same series), were pooled and processed as one sample in the cold. The tissue was washed 3 times with a buffer solution consisting of 0·15 mol/l KCl, 0·003 mol/l $NaHCO_3$ and 0·006 mol/l EDTA (pH 6·7) and homogenized with 2 ml of the buffer in an all-glass homogenizer. Two ml of 1 N perchloric acid (PCA) were added to the homogenate. After 30 min at 4°C, the homogenate was centrifuged for 10 min at 3000 rev/min and the resultant precipitate was washed twice with 0·5 N PCA and recentrifuged at the same speed. The washed precipitate was suspended in 1 N NaOH, shaken in a boiling water bath for 10 min and centrifuged at 3000 rev/min, for 10 min.

An aliquot of the supernatant was taken for counting the radioactivity as reported before (Finkelstein *et al.*, 1975) and in another aliquot the protein content was estimated by the method of Lowry *et al.* (1951). The synthesis of DNA and RNA was calculated by the incorporation of $^{3}$H-thymidine and $^{14}$C-uridine into DNA and RNA respectively per mg of protein. The effect of the steroids on the synthesis was calculated as percent of the mean " control ". The range between 90 and 110% was considered as " no effect ".

*Histopathological examination.* — From each tumour 3–4 randomly selected explants were examined histologically before organ culture in order to confirm the diagnosis of adenocarcinoma. Randomly selected explants were also examined after organ culture to further assess the viability of the tissue in culture. The explants were fixed in a 4% solution of neutral buffered formalin. After paraffin embedding, sections of 6 μm were cut and stained with haematoxylin and eosin and examined microscopically. When the nuclear morphology was preserved without pycnosis and when the nuclei stained normally with haematoxylin, the tissue was regarded as viable. Regressive changes were determined by varying degrees of pycnosis of the nuclei, karyorrhexis and faint or no staining of the nuclei with haematoxylin.

Statistical comparisons were performed by the Wilcoxon matched pairs signed-rank test (Siegel, 1956).

### RESULTS

### In vivo *experiments*

Ten animals, 7 with one tumour each, 2 with 2 tumours each and one animal with 3 tumours were ovariectomized (Table I). Of the 14 tumours examined, 10 regressed following ovariectomy and 4 did not. In 3 cases (No. VIII, IX, X), tumours in the same animal responded differently to the ovariectomy. Six of the animals whose tumours regressed after ovariectomy were injected with oestradiol-17β. This caused 4 tumours to grow, 2 continued to regress and in one tumour no clear effect was obtained.

TABLE I.—*The Effect* in vivo *of Castration and of Oestradiol-17β Administration on Rat DMBA Induced Adenocarcinoma*[1]

| Animal and tumour no. | Effect of castration | Effect of oestradiol-17β* |
|---|---|---|
| I | R | P |
| II | R | P |
| III | R | P |
| IV | R | R |
| V | R | R |
| VI | R | — |
| VII | P | — |
| VIII a | R | P |
| VIII b | R | NE |
| IX a | R | — |
| IX b | P | — |
| X a | P | — |
| X b | P | — |
| X c | R | — |

R, regression; P, progression; NE, no effect; —, not injected.
* s.c. 5 μg/day for 3–4 weeks.

Nine intact animals with 11 adenocarcinomata (2 animals having 2 tumours each) were injected with calusterone. Of the 11 tumours examined, 8 regressed following calusterone administration. The 3 tumours which did not regress were unusually big (40, 46 and 47 mm mean diameter) when the injections were started. Figure 1 shows the tumour diameter curves (mean ± s.e.) of animals treated with calusterone compared with 6 controls.

### *Organ culture experiments*

The effects of calusterone, testosterone and oestradiol-17β on explants of rat adenocarcinoma in organ culture are shown in Table II. Calusterone inhibited the synthesis of DNA in all 7 cases examined ($P \leqslant 0{\cdot}02$) and the synthesis of RNA was also inhibited in 6 of 7 cases ($P \leqslant 0{\cdot}05$). Testosterone inhibited the synthesis of DNA in 12 of 14 cases and the RNA synthesis was inhibited in 10 cases ($P < 0{\cdot}01$ in both tests). Oestradiol-17β inhibited the synthesis of DNA in 6 of 10 cases, had no effect in 3 and enhanced the synthesis of DNA in one case. RNA synthesis was inhibited by oestradiol-17β in 5 cases and stimulated in 2. (N.S. in both.)

All tissue samples grown in organ culture in a steroid-free medium and most samples grown with testosterone and oestradiol-17β had a vital appearance. Conversely, all samples in which calusterone was added to the medium underwent complete or partial regressive changes.

### DISCUSSION

The effect of calusterone on rat adenocarcinoma in organ culture and *in*

TABLE II.—*Effects of Calusterone, Testosterone and Oestradiol-17β on the Synthesis of DNA and RNA in Organ Culture of Rat Adenocarcinoma*[1]

| | $^3$H-Thymidine incorporation into DNA | | | | $^{14}$C-Uridine incorporation into RNA | | | |
|---|---|---|---|---|---|---|---|---|
| | | Calust. | Test. | $E_2$ | | Calust. | Test. | $E_2$ |
| Exp. No. | Cont. ± s.e. ct/min/µg prot. | % incorporation of cont. | | | Cont. ± s.e. ct/min/µg prot. | % incorporation of cont. | | |
| 1 | 84±13·0 | — | 71 | 95 | 10± 1·6 | — | 104 | 86 |
| 2 | 39± 3·1 | — | 40 | 75 | 5± 0·6 | — | 70 | 80 |
| 3 | 116±17·5 | — | 12* | 36 | 34± 2·7 | — | 45 | 90 |
| 4 | 140± 9·4 | — | 69* | 85 | 8± 1·1 | — | 110 | 105 |
| 5 | 314± 8·6 | — | 62 | 120 | 26± 4·2 | — | 76 | 126 |
| 6 | 128± 9·2 | — | 30 | 87 | 39± 3·5 | — | 80 | 140 |
| 7 | 78± 0 | — | 40 | 83 | 6± 0 | — | 80 | 81 |
| 8 | 69± 7·8 | 11† | 93 | 101 | 18± 2·0 | 45 | 104 | 95 |
| 9 | 79±12·0 | 67* | 94 | — | 24± 2·3 | 37 | 75 | — |
| 10 | 143±18·0 | 46† | 80 | 58* | 8± 1·3 | 105 | 93 | 68 |
| 13 | 55± 5·8 | 60† | 87 | — | 29± 3·6 | 64 | 79 | — |
| 14 | 79± 5·3 | 50* | 57 | — | 27± 1·9 | 71 | 73 | — |
| 15 | 22± 3·4 | 10* | 81 | 105 | 9± 0·5 | 31 | 87 | 66 |
| 16 | 125±15·7 | 30* | 64 | — | 77±10·6 | 44 | 89 | — |
| Mean | | 39·1 | 62·8 | 82·0 | | 56·7 | 83·2 | 96·1 |
| S.D. | | 22·7 | 24·7 | 24·3 | | 25·6 | 16·7 | 22·3 |
| Statistical significance | | $P \leqslant 0·02$ | $P < 0·01$ | N.S. | | $P \leqslant 0·05$ | $P < 0·01$ | N.S. |

Cont., control; Calust., calusterone; Test., testosterone; $E_2$, oestradiol-17β. Prot., protein. N.S. Not significant.

* Partial regressive changes.

† Complete autolysis.

FIG. 1.—Percent change (mean ± s.e.) in tumour diameter following calusterone administration. ——— Controls injected with 0·2 ml sterile vehicle daily (6 tumours). Initial tumour diameter 15–25 mm (= 100%). - - - - - Calusterone injected 10 mg/0·2 ml sterile vehicle daily (8 tumours). Initial tumour diameter 15–25 mm (= 100%). – – – – Calusterone injected 10 mg/0·2 ml sterile vehicle daily (3 tumours). Initial tumour diameter 40–47 mm (= 100%).

*vivo* were compared. Using the technique of organ culture makes it possible to study the direct effect of a hormone on the tissue. Although the organ culture conditions cannot be regarded as physiological, they resemble the physiological conditions much more than any other *in vitro* technique. The testosterone derivative, calusterone, had an anti-tumour effect in all the 7 tumours studied in organ culture. Not only did it strongly inhibit the incorporation of $^3$H-thymidine into DNA and to a lesser extend also of $^{14}$C-uridine into RNA ($P \leqslant 0{\cdot}02$ and $\leqslant 0{\cdot}05$ respectively, because only 7 samples were examined) but histologically regressive changes appeared after organ culture whereas the " controls " and most of the samples with added oestradiol-17$\beta$ or testosterone retained their vital appearance. Administration of calusterone *in vivo* caused regression of all 8 tumours with an initial mean diameter of 30 mm or less. In 3 tumours with a mean diameter of 40, 46 and 47 mm calesterone had no effect *in vivo*. This is in agreement with the report of Griswold and Green (1970) who found tumour regression after androgen administration (2$\alpha$-methyl-dihydrotestosterone propionate) only in those with a weight of 0·3–0·5 g whereas tumours with a weight of 5–10 g did not regress following androgen administration.

The anti-tumour effect of calusterone both in organ culture and *in vivo* is very interesting in view of the results obtained in breast cancer patients and in animal experiments *in vivo*. Gordan *et al.* (1973) found that 200 mg/day orally of calusterone produced objective regressions in 51% of patients with advanced breast cancer compared with only 21·5% after testosterone propionate and 16% after stilboestrol. On the other hand, subcutaneous administration of calusterone 5 mg/kg/day for 10 days to 6 Sprague–Dawley rats with R-35 mammary adenocarcinoma had no anti-tumour effect (Booth, 1968). Since in this publication it was reported that calusterone is not toxic to rats in amounts less than 68 mg/kg/day, we have chosen to inject the animals with 10 mg of calusterone daily (40 mg/kg/day) as reported by Segaloff (1966) for testosterone propionate. The difference in the *in vivo* results with rat adenocarcinoma could be due to a different tumour used in a different strain of rats. In view of the report of Kim (1965) who injected 0·1, 1·0 and 10·0 mg of testosterone propionate into female rats with adenocarcinoma and found that all 3 doses had an inhibitory effect on tumour growth, and lowest dose had the strongest effect, it seems that the different amount of calusterone used by us was not necessarily the cause of the different results. Our experimental model with DMBA-induced adenocarcinoma in Lewis female rats apparently correlates better with the *in vivo* results obtained in human breast cancer patients as described by Gordan *et al.* (1973).

In contrast to calusterone which had an anti-tumour efficacy both in organ culture and *in vivo*, oestradiol-17$\beta$ had practically no effect on the synthesis of DNA and RNA in organ culture, as was also reported by Welsch and Rivera (1972) for oestradiol-17$\beta$ at concentrations of 0·0001–1·0 $\mu$g/ml. However, in our experiment as well as in those of others (Huggins *et al.*, 1959; Kim and Furth, 1960; Teller *et al.*, 1969) 70% of the tumours were ovarian dependent. This could be explained by the assumption that calusterone acts directly on the mammary adenocarcinoma, as judged by the same effect obtained *in vivo* and in organ culture. Oestradiol-17$\beta$, on the other hand, may exert its effect indirectly by activating other hormones such as prolactin which acts on the tumour. A variety of experiments have indicated that oestrogens are mammary oncogenic primarily as a result of their ability to influence prolactin secretion (Sterental *et al.*, 1963; Kim, 1965; Meites and Nicoll, 1966; Klaiber *et al.*, 1969; Welsch and Rivera, 1972). In the *in vivo* experiment, where the animals were castrated, regression occurred because of the absence of ovarian hormones to stimulate the secretion of prolactin. Following regression of tumours, the animals were injected with 5 $\mu$g daily of oestradiol-17$\beta$. This is a relatively small dose chosen to reverse the effect of ovariectomy, as described by Kim (1965) and McGuire and Julian (1971). Larger doses of oestradiol-17$\beta$, *e.g.* 30 $\mu$g (Kim, 1965) or 1200–1500 $\mu$g daily (Teller *et al.*, 1969) could have been inhibitory.

The correlation between the effect on adenocarcinoma *in vivo* and in organ culture of other steroid hormones is being investigated.

We thank Drs R. S. Swain and J. C. Babcock from the Upjohn Company, Kalamazoo, Mich., for making calusterone available to us.

This work was supported by a grant from the Israel Cancer Association No. 10/75.

This study is part of a thesis to be submitted by I. Erlichman to the Hebrew University in partial fulfillment of the requirements for the M.Sc. degree.

### REFERENCES

Booth, S. (1968) 7$\beta$,17-Dimethyltestosterone (NSC 88536). Clinical brochure. Bethesda: Endocrine Evaluation Branch, National Cancer Institute. p. 1.

Finkelstein, M., Geier, A., Horn, H., Levij, I. S. & Ever-Hadani, P. (1975) Effect of Testosterone and Estradiol-17$\beta$ on Synthesis of DNA, RNA and Protein in Human Breast in Organ Culture. *Int. J. Cancer*, **15**, 78.

Gordan, G. S., Halden, A., Horn, Y., Fuery, J. J., Parsons, R. J. & Walter, R. M. (1973) Calusterone (7$\beta$,17$\alpha$-dimethyltestosterone) as Primary and Secondary Therapy of Advanced Breast Cancer. *Oncology*, **28**, 138.

Griswold, D. P. & Green, C. H. (1970) Observation on the Hormone Sensitivity of 7,12-dimethylbenz($\alpha$)anthracene-induced Mammary Tumors in the Sprague–Dawley Rat. *Cancer Res.*, **30**, 819.

Heise, E. & Gorlich, M. (1966) Growth and Therapy of Mammary Tumours Induced by 7,12-dimethylbenzanthracene in Rats. *Br. J. Cancer*, **20**, 539.

Huggins, C., Braziarelli, G. & Sutton, H. (1959) Rapid Induction of Mammary Carcinoma in the Rat and the Influence of Hormone on the Tumor. *J. exp. Med.*, **109**, 25.

Huggins, C., Grand, L. C. & Brilliantes, F. P. (1961) Mammary Carcinoma Induced by a Single Feeding of Polynuclear Hydrocarbon and its Suppression. *Nature, Lond.*, **189**, 204.

Kim, U. (1965) Pituitary Function and Hormonal Therapy of Experimental Breast Cancer. *Cancer Res.*, **25**, 1146.

Kim, U. & Furth, J. (1960) Relation of Mammary Tumors to Mammotropes. II. Hormone Responsiveness of 3-methylchlolanthrene Induced Mammary Carcinomas. *Proc. Soc. exp. Biol. Med.*, **103**, 613.

Klaiber, M. S., Gruenstein, M., Meranze, D. R. & Shimkin, M. B. (1969) Influence of Hypothalamic Lesions on the Induction and Growth of Mammary Cancers in Sprague–Dawley Rats receiving 7,12-dimethylbenz($\alpha$)anthracene. *Cancer Res.*, **29**, 999.

Lowry, O. H., Rosebrough, N. J., Farr, A. L. & Randall, R. J. (1951) Protein Measurement with the Folin Phenol Reagent. *J. biol. Chem.*, **193**, 265.

Meites, J. & Nicoll, C. S. (1966) Adenohypophysis: Prolactin. *A. Rev. Physiol.*, **28**, 57.

McGuire, W. L. & Julian, J. A. (1971) Comparison of Macromolecular Binding of Estradiol in Hormone-dependent and Hormone-independent Rat Mammary Carcinoma. *Cancer Res.*, **31**, 1440.

Segaloff, A. (1966) Hormones and Breast Cancer. *Rec. Prog. Horm. Res.*, **22**, 351.

Siegel, S. (1956) *Nonparametric Statistics for the Behavioural Sciences.* New York: McGraw-Hill Book Co. p. 75.

Sterental, A., Dominguez, J. M., Weissman, C. & Pearson, O. H. (1963) Pituitary Role in the Estrogen Dependency of Experimental Mammary Cancer. *Cancer Res.*, **23**, 481.

Teller, M. N., Kaufman, R. J., Bowie, M. & Stock, C. C. (1969) Influence of Estrogens and Endocrine Ablation on Duration of Remission Produced by Ovariectomy or Androgen Treatment of 7,12-dimethylbenz($\alpha$)anthracene-induced Rat Mammary Tumors. *Cancer Res.*, **29**, 349.

Welsch, C. W. & Rivera, E. M. (1972) Differential Effects of Estrogens and Prolactin on DNA Synthesis in Organ Culture of DMBA-induced Rat Mammary Carcinoma. *Proc. Soc. exp. Biol. Med.*, **139**, 623.